# Math Fluency during Primary School

**DOI:** 10.3390/brainsci12030371

**Published:** 2022-03-11

**Authors:** Yarden Gliksman, Shir Berebbi, Avishai Henik

**Affiliations:** 1 Department of Behavioral Sciences, Ruppin Academic Center, Emek Hefer 4025000, Israel; 2 Department of Psychology and Zlotowski Center for Neuroscience, Ben-Gurion University of the Negev, Beer-Sheva 8410501, Israel; shirb300@gmail.com

**Keywords:** math fluency, math facts, calculation and retrieval, arithmetic in primary school, arithmetic operations

## Abstract

Math fluency is the ability to solve arithmetic facts quickly and accurately (i.e., addition and subtraction problems up to 20, and multiplication and division problems from the multiplication table). Curricula in primary school devote a significant period of time for learning and retrieval of arithmetic facts. Recently, a new computerized tool to assess math fluency—the BGU-MF (Ben-Gurion University Math Fluency) test—was developed and found to be a reliable and valid tool for adults. In the current study, we examine the performance of first to sixth-grade children in math fluency using the BGU-MF. The results present the performance of MF during childhood and emphasize that it continues to develop during primary school. Importantly, proficiency of MF differed by operations, and the automaticity of math facts was acquired in different grades. Moreover, we found that the BGU-MF is a reliable and valid tool not only for adults but also for children during primary school. Our study has educational implications for the teaching, practice, and retrieval of arithmetic facts.

## 1. Introduction

Using numbers is an integral part of our life. We use numbers when we measure in cooking, find a page in a book, during sports activities, and so on. Specifically, simple calculations are an everyday activity, in adults’ life (e.g., calculating the number of invited guests for a family dinner) and in children’s life (e.g., calculating their pocket money). Understanding numbers and performing simple calculations efficiently are the building blocks of quantitative reasoning [1], and they were found to predict academic success and level of income [2,3]. Accordingly, the math education curriculum during primary school devotes a great deal of time to learning, practicing, memorizing, and retrieving simple arithmetic problems, known as math facts. Math facts are the basis for more advanced arithmetic, such as solving equations and verbal questions, carrying over problems, etc.

Math facts are problems with operands from 0 to 10 with the four basic arithmetic operations: addition and subtraction, with answers between 0 to 20 (e.g., 9 + 5; 16 − 7), and multiplication and division from the multiplication table (e.g., 6 × 5; 45 ÷ 9). In Israel and in other OECD (Organisation for Economic Co-operation and Development) countries, the arithmetic curriculum relates specifically to the learning of math facts. The first-grade curriculum includes learning addition and subtraction up to 20. The second grade includes more practice and memorization of addition and subtraction math facts, and by the end of the second grade, multiplication is taught as a repeated addition. In many cases, during the second grade, only multiplications with operands between 1 and 5 are covered. During the third grade, the full multiplication table is taught, as is the division operation. During the fourth grade, a mastery of all math facts for all operations should be attained [4]. In light of the time devoted to teaching math facts during primary school, how proficient are those students with math facts? A possible way to explore this question is by studying math fluency.

Math fluency is the ability to proficiently solve math facts, accurately and quickly. Math fluency was found to be a stable ability [5,6] and to predict math achievements throughout the school years of primary school students [7,8,9], high school students [10], and college students [11]. During the first school years, math fluency improves with learning and practice, and use of strategy [12]. Strategies change with development. First, math facts are solved by using fingers or material objects and a “counting all” strategy. Then, children count up from one of the operators in the problem, using the “counting-on strategy” (e.g., 4 + 3 counts as 4-5-6-7). Next, children use arithmetic principles and knowledge of other combinations, and use decomposition strategies such as knowledge of related facts or decomposing numbers into parts (e.g., 7 + 5 = 7 + 3 + 2). Finally, children retrieve the solutions from long-term memory [13,14]. What is the relation between arithmetic principles and retrieval? Different observations have been suggested. Siegler [15], following Piaget [16], suggested that retrieval is achieved after the repetitive practice of each combination (e.g., 3 + 2 = 5) and that children use the complementarity principle to solve facts from the same combination. Namely, in order to solve a subtraction fact (e.g., 12 − 4), children use their understanding that subtraction is the inverse of addition (e.g., 4 + ? = 12). Thus, a complementary relation provides a basis for fast and accurate production of math facts (i.e., retrieval). In contrast, Baroody [17,18] suggested that children use multiple strategies to answer math facts and that the understanding of the relation between opposite operations may be accomplished with practice, even after retrieval occurs. Regarding math fluency, according to Siegler’s observation, the retrieval of math facts of opposite operations (i.e., addition-subtraction; multiplication-division) should be accomplished at the same time during development. However, according to Baroody’s observation, retrieval of math facts of different operations may be accomplished at different developmental stages.

Despite its importance, only a few math fluency evaluations tools have been developed (e.g., Tempo Test Rekenen [19], Math racer [20]). The most common test of math fluency is a subtest in the Woodcock–Johnson [WJ] battery [21], which is a paper and pencil test, and applied manually. Importantly, several issues should be taken into consideration when using the WJ test. The WJ does not include division assessment, and the distribution of the addition, subtraction, and multiplication operations is not equal in the test, as multiplication appears only from problem #61 and on. Moreover, the measures in the tests are total accuracy rates and the number of correctly solved problems, and it does not enable assessing the performance of each operation separately. Of significant note is that, as the WJ is applied manually, it does not enable evaluating performance in measures of response times (RTs). Last, the total number of problems is fixed (i.e., 151), thus it might cause a ceiling effect and not distinguish sensitively enough between participants with high math abilities (e.g., gifted children).

Recently, the BGU-MF (Ben-Gurion University Math Fluency) test was developed. It is a simple, computerized, timed test designed to evaluate math fluency. The BGU-MF test (henceforth the BGU-MF) includes math facts of all four operations. Moreover, as it is computerized, the output includes a measure of RT for each problem separately. The measure of RT is important as it examines efficacy in math fluency beyond accuracy. Moreover, it enables to distinguish between the performance of different operations. In an earlier study, the BGU-MF was found to be reliable and valid for adults [5].

Following Gliksman et al. [5], we had two major aims: to test the reliability and validity of the BGU-MF and to study resultant changes in math fluency in primary school in relation to curriculum math facts taught during these grades. With respect to testing the BGU-MF, we had three goals: (1) Examine the reliability of the BGU-MF in primary school, from first to sixth grade. Thus, each child performed the BGU-MF twice in different sessions, and their performance was compared. Following previous studies, we hypothesized the BGU-MF would be found to be reliable also for children [5,6]. (2) Examine the validity of the BGU-MF in primary school. Thus, each child additionally performed the paper and pencil WJ math fluency test for comparison. Following Gliksman et al. [5], we hypothesized that a correlation between the two formats of math fluency would be found. The purpose for testing reliability and validity is so that the BGU-MF may be used in future research and for interventions; (3) Examine the relation between math fluency (via the BGU-MF) and other math abilities. Thus, half of the participants performed other subtests of the arithmetic battery of WJ. We hypothesized that performance in the BGU-MF would correlate with the WJ verbal questions subtest, which required transformation of a situation into an equation. Regarding the other arithmetic subtests, following Gliksman et al. [5] who found that in adults, BGU-MF performance correlated with that in the math matriculation exam, we predicted that a correlation with the BGU-MF would be found; however, it might be weaker.

Additionally, we aimed to describe changes in the performance of math fluency during primary school; namely, to compare the accuracy and RTs for different operations, and the total corrected number of solved problems in the BGU-MF, between grade levels. We expected that all measures of fluency would improve with age and education.

## 2. Materials and Methods

### 2.1. Participants

One hundred and twenty-two children from primary school (72 females, aged 6- to 11.5-years old, average = 9.3, SD = 1.6) took part in the experiment. The number of children in each grade from the first to sixth grade was: 16 (8 female), 16 (10 female), 24 (17 female), 23 (11 female), 23 (13 female), and 20 (13 female), respectively. The participants were recruited from four different localities in Israel, all of them from middle-high socio-economic status. Participants were naive to the purpose of the study. Fifty-five participants performed the full battery of the experiment (two sessions of the BGU-MF, and the full WJ math battery). An additional 67 participants performed two sessions of the BGU-MF and one test of the WJ battery—the math fluency subtest. All participants and their parents gave their informed consent prior to participation in the study. The study was carried out following the guidelines of the protocol approved by Ben-Gurion University of the Negev’s Ethics Committee.

### 2.2. Procedure

The procedure included two sessions. Each participant performed the BGU-MF in each session, in a quiet room. In one of those sessions, they performed either the full WJ arithmetic battery or part of it. The order of sessions and the order of tests (BGU-MF first, WJ first) were counterbalanced across participants. The gap between the sessions was between one week and 10 days. Data were collected during 2018–2020, between November and February each year.

#### 2.2.1. The BGU-MF (Ben-Gurion University Math Fluency)

**Figure 1:**
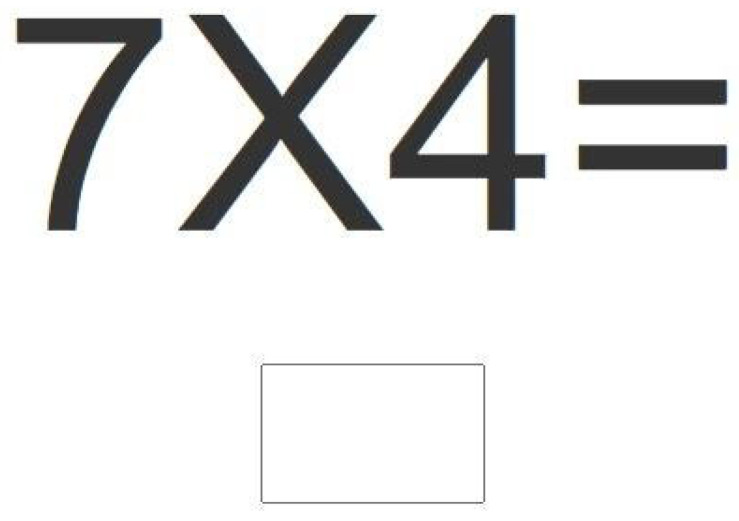
An example of a problem from the BGU-MF.

The BGU-MF is a computerized test for examining math fluency. The test includes math facts of the four basic operations: addition and subtraction (with answers or operators up to 20; e.g., 14 − 7), and multiplication and division taken from the multiplication table (up to 10 × 10; e.g., 4 × 8). Problems are selected randomly from the full set of trials (see Supplementary Materials for the full set of problems). The problems appeared one by one on a screen, and participants were instructed to type in their answers inside a square and press the Enter key in order to proceed to the next trial (see Figure 1). The length of the test was 180 s. Participants were instructed to answer as accurately and quickly as possible. For each participant, we calculated the number of correctly solved problems, accuracy rates, and RTs for correct responses. The RTs were calculated from the moment the problem appeared on the screen until the first digit of the solution was typed on the keyboard. Importantly, the BGU-MF may be modified by the experimenter, who can decide which operations will be included in the test, enabling adjustment for relevant class and curriculum (e.g., first-grade students would not be examined using division exercises), and whether the exercises will include the operand zero or not (for further description of the BGU-MF, see Gliksman et al. [5]). In the current study, only addition and subtraction operations were included for participants in the first and second grade. For participants in the third to sixth grade, all operations were included in the test. The full code for the computerized math fluency tool and instructions for its modification are available online at https://github.com/YardenGliksman/BGU-MF (accessed on 12 January 2022).

#### 2.2.2. Woodcock Johnson Battery

The math WJ battery included five subtests. In all subtests, the measure of performance was accuracy rates (for a full description of the tests see Woodcock et al. [21]).

##### Paper and Pencil Math Fluency

The test included a table of 151 addition, subtraction, and multiplication math facts. Participants were instructed to write the answer as accurately and quickly as possible. The length of the test was 180 s. Performance was measured by accuracy rates and the number of correctly solved problems.

##### Sequence Completion

The test included 24 series. The participants were instructed to fill in the missing number in the series (e.g., 7, 14, 28, __). The difficulty level was increased as the task progressed.

##### Applied Problems

The test included 63 text problems. The problems included reading an analog clock (e.g., “point to the clock that shows 7 o’clock”), conversion and itemization of money (e.g., “4 people have 6 dollars each, how much money do they have altogether?”), calculation skills (e.g., “Look at the squares. If you paint 4 more squares, how many squares will there be?”), geometric knowledge (e.g., “calculate the perimeter of the polygon”), etc.

##### Quantitative Concept

The test included 34 problems of formal knowledge, such as symbol recognition (e.g., “recognize the plus (+) symbol”), etc.

##### Calculation

The test included 35 problems regarding the use of computational procedures. The problems included the four basic operations and advanced arithmetic calculation, such as subtraction of fractions (e.g., $\frac{2}{3}-\frac{1}{3}$).

## 3. Results

The analysis included an examination of the reliability and validity of the BGU-MF in children in primary school. Following this, we report the performance of math fluency in each grade. All reported planned comparisons are after Bonferroni corrections. All analyses reported are for correct responses only.

### 3.1. Reliability of the BGU-MF

To examine the reliability, we compared the performance of two sessions of the BGU-MF on measures of the number of solved problems, accuracy, and RTs. We performed paired samples *t*-tests, correlations, and Cronbach alpha analyses. We report the Pearson correlation, but if a ceiling effect occurred, then a Spearman correlation is reported. These analyses included data from all the 122 participants.

#### 3.1.1. Number of Solved Problems

**Figure 2:**
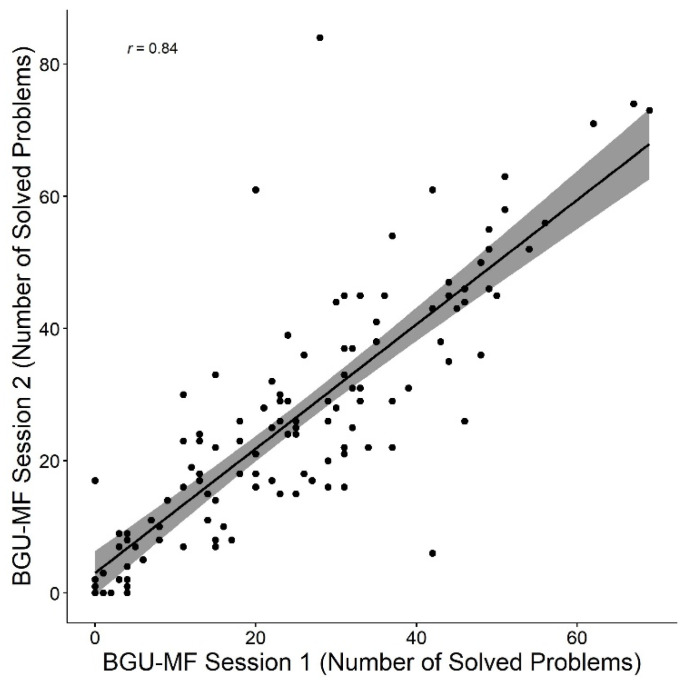
Correlation between number of solved problems in the first and second sessions in the BGU-MF.

The mean number of solved problems in 180 s was 25.3 (*SD* = 16.8). Analysis of paired samples *t*-tests revealed no differences between the two sessions: *t* (121) = −1.2, *p* = 0.26, *d* = −0.1. The correlation between sessions was significant, *r* = 0.84, *p* < 0.001 (see Figure 2). Last, the calculation of Cronbach’s alpha for the number of solved problems was 0.94 (the calculation was based on the number of problems performed in each of six parts of 30 s each). Taken together, the reliability of the BGU-MF was supported in terms of the measure of the number of solved problems.

#### 3.1.2. Accuracy

**Figure 3:**
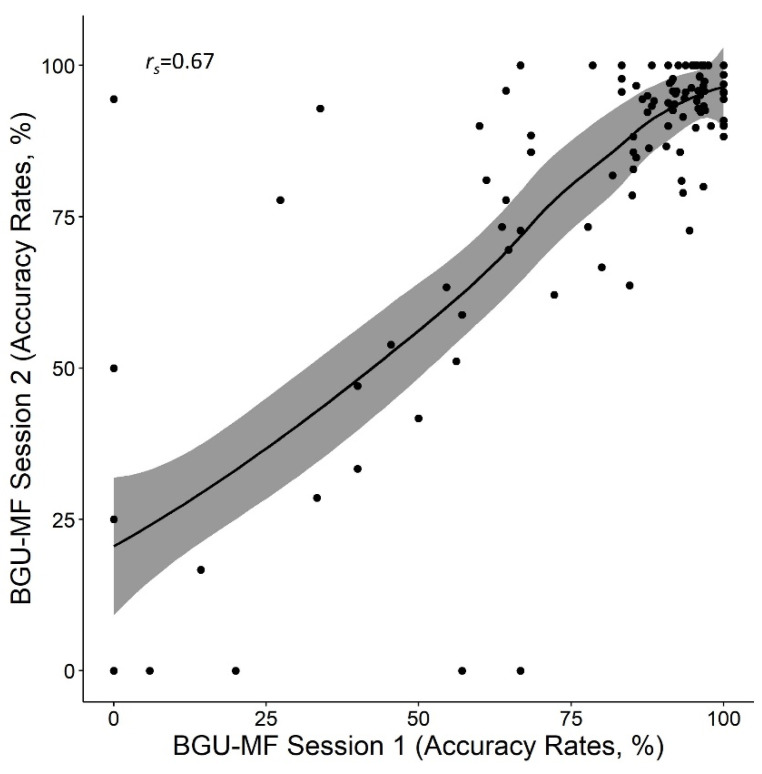
Correlation between accuracy rates in the first and second sessions in the BGU-MF.

The mean accuracy rate was 80.1% (*SD* = 26.3). The analysis of paired samples *t*-test revealed no differences between the two sessions: *t* (120) = −1.6, *p* = 0.10, *d* = 0.15. The Spearman correlation between sessions was significant, *r_s_* = 0.67, *p* < 0.001 (see Figure 3). Note that a ceiling effect appears, as many participants achieved 100% accuracy in one of the sessions (15% of participants in the first session and 20% of participants in the second session). Cronbach’s alpha was 0.89. Taken together, the reliability of the BGU-MF was supported in terms of the measure of accuracy rates.

#### 3.1.3. RTs

**Figure 4:**
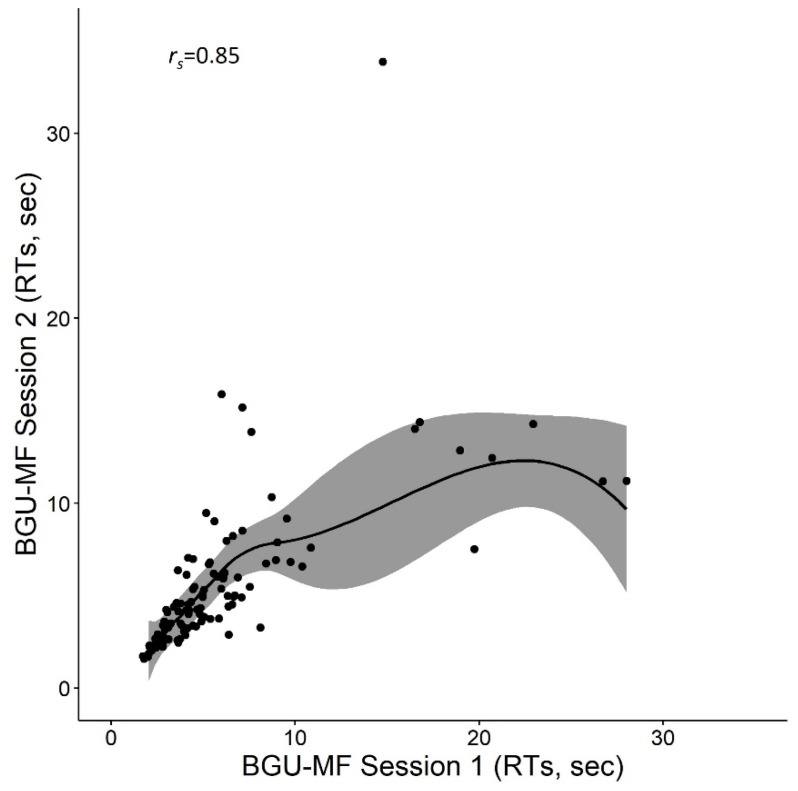
Correlation between RTs in the first and second sessions in the BGU-MF.

The average RTs for correct responses per problem was 6.9 s (*SD* = 8.1). Fifteen participants from the first grade (7 in the first session and 8 in the second session) were excluded from the correlation analysis because they did not have any correct responses in one of the sessions. Analysis of paired samples *t*-test revealed no differences between the two sessions: *t* (109) = 1.2, *p* = 0.2, *d* = 0.12. The Spearman correlation between sessions was significant, *r_s_* = 0.85, *p* < 0.001 (see Figure 4). Cronbach’s alpha was 0.79. Taken together, the reliability of the BGU-MF was supported in terms of the measure of RTs.

#### 3.1.4. Split Test Reliability of the BGU-MF

The BGU-MF is relatively short. However, in order to examine whether learning or fatigue effects occurred during the test, we compared the first half of the test (0–90 s) with the second half (91–180 s). We found a significant correlation between halves for all measures, *r* = 0.75; 0.64; 0.65; *p* < 0.001 for all comparisons, number of solved problems, accuracy, and RT, respectively.

We also performed paired samples *t*-tests for first vs. second halves with the number of problems, accuracy rates, and RTs as dependent variables. For the number of corrected problems, the analysis revealed no differences between halves, *t* < 1. For accuracy rates and RTs, the analysis revealed significant differences between halves, as participants become more accurate and faster in the second halves, *t* (227) = 1.9, *p* = 0.05, *d* = −0.13; *t* (212) = 2.9, *p* < 0.01, *d* = 0.19, for accuracy and RT, respectively. For an analysis of variance (ANOVA) of halves and grades for all measures, see Supplementary Materials. These results again strengthen our finding that the BGU-MF is a reliable test but indicate that familiarization contributed to better performance during the test.

### 3.2. Validity: Comparison between Math Fluency Formats

**Figure 5:**
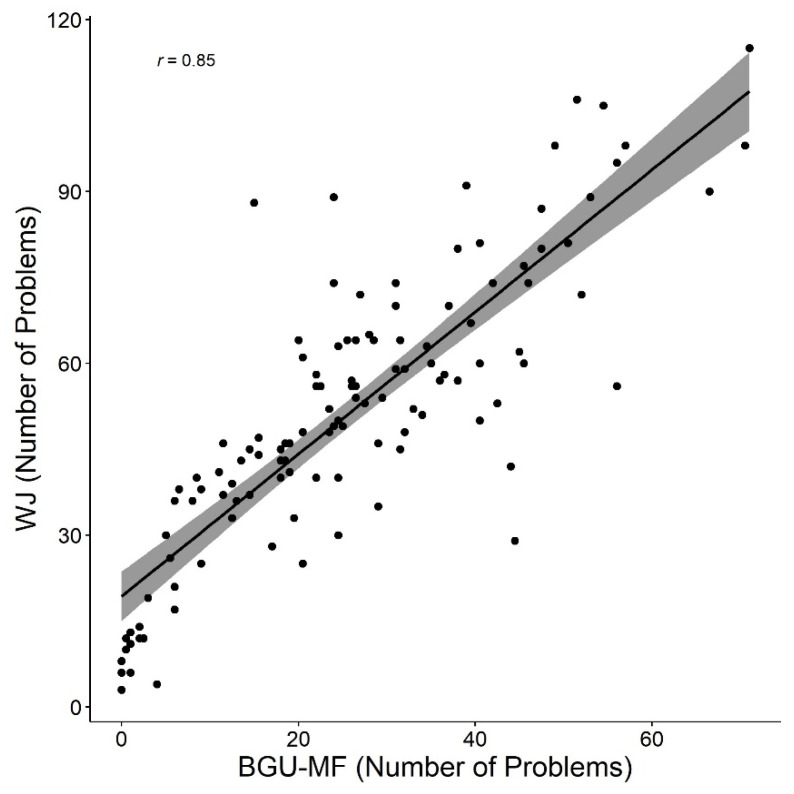
Number of solved problems in both formats of the math fluency tests.

**Figure 6:**
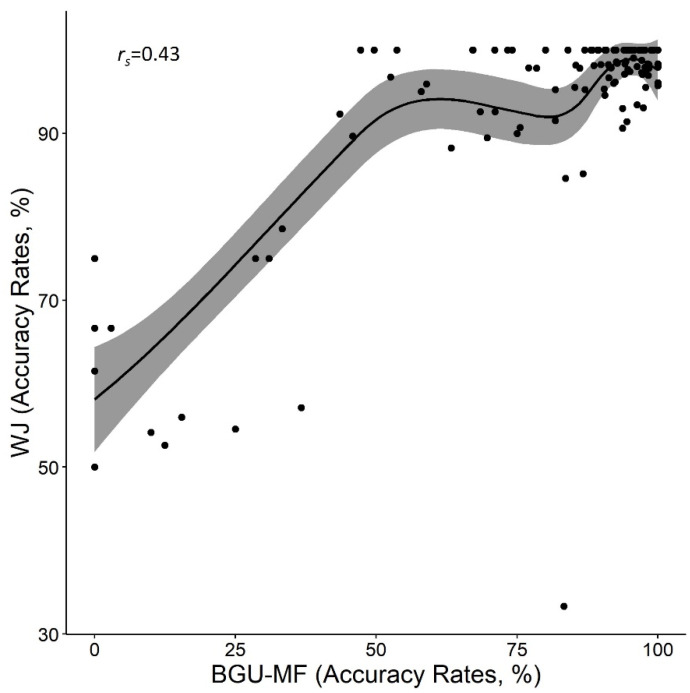
Accuracy rates in both formats of the math fluency tests.

In order to examine the validity of the BGU-MF, we compared the average performance of math fluency in the BGU-MF across the two sessions with that of the WJ paper and pencil math fluency test (WJ in what follows). An analysis of paired samples *t*-test was carried out with the number of solved problems and accuracy rates as dependent measures. The WJ format was easier for participants on both measures: the number of solved problems was 50.9 (*SD =* 24.6) vs. 25.5 (*SD =* 16.8), for WJ and BGU-MF, respectively, *t* (121) = 20.5, *p* < 0.001, *d* = 1.9; accuracy rates were 93.3% (*SD =* 12.7) vs. 80.1% (*SD* = 26.3), for WJ and BGU-MF, respectively, *t* (121) = 7.8, *p* < 0.001, *d* = 0.07. The correlations between the performance in the two formats were significant, *r* = 0.85, and *r_s_* = 0.43, *p* < 0.001 for all comparisons, for the number of problems and accuracy rates, respectively (see Figure 5 and Figure 6, Table 1). Note, that in Figure 6, a ceiling effect appears, as quite a few participants achieved 100% accuracy in one of the formats but presented changes in accuracy in the other session. See additional analysis regarding differences between formats and grades in the Supplementary Materials.

### 3.3. Validity: Comparison between the BGU-MF and the Other Math Test in the WJ Battery

Correlations between measures of the BGU-MF (number of solved problems, accuracy rates, and RTs) and the accuracy in the WJ subtests were calculated and found to be medium-to-high for all subtests except quantitative concepts (see Table 1), namely, math fluency related to the performance of other math abilities.

### 3.4. Math Fluency during Primary School

In this part, we present the BGU-MF performance across the different grades in measures of the number of solved problems, accuracy rates, and RTs. A one-way ANOVA was carried out with grade as a between-subjects independent variable. For the measures of accuracy rates and RTs, we also carried out an ANOVA per operation and a comparison between operations. For multiplication and division, the analysis was carried out for third to sixth-grade students. Additionally, we calculated trends for each measure. The data per grade are presented in Table 2.

#### 3.4.1. Number of Solved Exercises

**Figure 7:**
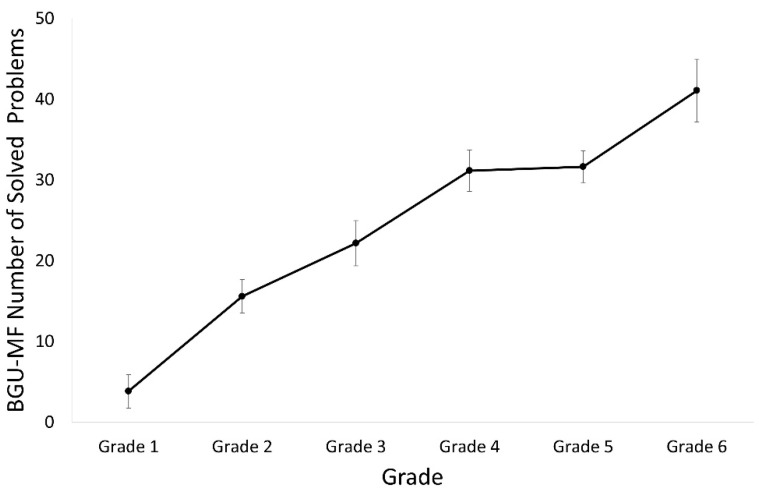
Number of solved problems in the BGU-MF for each grade.

A one-way ANOVA was carried out. The analysis revealed a main effect of grade, *F* (5, 116) = 20.1, *p* < 0.001, *η_p_*^2^ = 0.46. Planned comparisons revealed that the number of solved problems in the first grade was lower and significantly different from that of the third to sixth grade, *p* < 0.001 for all comparisons. The number of solved problems in the second grade was lower and significantly different from that in the fourth, fifth, and sixth grade, *p* = 0.003, *p* = 0.002, and *p* < 0.001, respectively. The number of solved problems in the third grade was lower and significantly different from that in the sixth grade, *p* < 0.001. A linear trend analysis revealed a significant linear trend, *t* (116) = 9.9, *p* < 0.001 (see Figure 7).

#### 3.4.2. Accuracy Rates

##### General Accuracy Rate

The analysis revealed a main effect of grade, *F* (5, 116) *=* 34.3, *p* < 0.001, *η_p_*^2^ = 0.6. A trend analysis revealed a significant linear trend, *t* (116) = 10.6, *p* < 0.001; a quadratic trend, *t* (116) = −7.9, *p* < 0.001; and a cubic trend, *t* (116) =2.9, *p* = 0.004.

##### Addition

The analysis revealed a main effect of grade, *F* (5, 116) *=* 23.0, *p* < 0.001, *η_p_*^2^ = 0.5. Planned comparisons revealed that the accuracy rate in the first grade was lower and significantly different from that in the second to sixth grade, *p* < 0.001 for all comparisons. No other differences were found. A trend analysis revealed a significant linear trend, *t* (116) = 8.1, *p* < 0.001; a quadratic trend *t* (116) = −7.1, *p* < 0.001; and a cubic trend, *t* (116) = 2.9, *p* = 0.004.

##### Subtraction

The analysis revealed a main effect of grade, *F* (5, 116) = 26.6, *p* < 0.001, *η_p_*^2^ = 0.53. Planned comparisons revealed that the accuracy rate in the first grade was lower and significantly different from that in the second to sixth grade, *p* < 0.001 for all comparisons. Accuracy rate in the second grade was lower and different from that in the fourth and fifth grade, *p* = 0.02, *p* = 0.04, *p* = 0.09, respectively. No other differences were found. A trend analysis revealed a significant linear trend, *t* (116) = 8.9, *p* < 0.001; a quadratic trend, *t* (116) = −7.6, *p* < 0.001; and a cubic trend, *t* (116) = 2.4, *p* = 0.017.

##### Multiplication

The analysis revealed no main effect of grade, *F* (3, 85) = 2.0, *p =* 0.13, *η_p_*^2^ = 0.07. A trend analysis revealed a significant quadratic trend, *t* (85) = −2.2, *p* = 0.03.

##### Division

**Figure 8:**
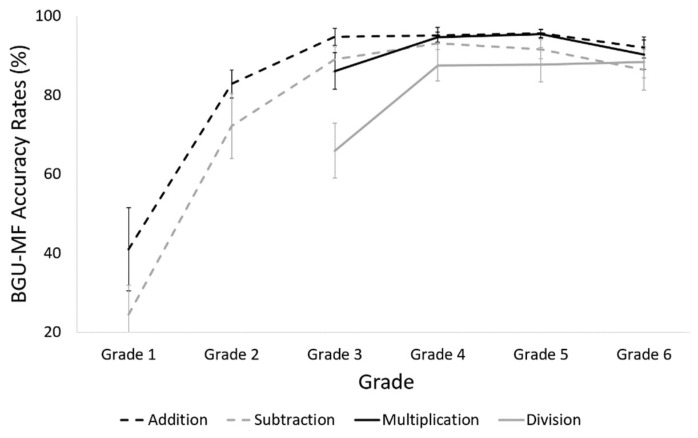
Accuracy rates in the BGU-MF, of every operation, in each grade.

The analysis revealed a main effect of grade, *F* (3, 85) = 4.5, *p* = 0.005, *η_p_*^2^ = 0.14. Planned comparisons revealed that the accuracy rate in the third grade was lower and significantly different from that in the fourth to sixth grade, *p* = 0.02 for all comparisons. No other differences were found. A trend analysis revealed a significant linear trend, *t* (85) = 2.9, *p* = 0.005, and a quadratic trend, *t* (85) = −2.0, *p* = 0.05 (see Figure 8).

##### Comparison between Operations

The analysis revealed a main effect of operation, *F* (3, 264) = 10.1, *p* < 0.001, *η_p_*^2^ = 0.1. Planned comparisons revealed that the accuracy rate of division was lower and significantly different from that of addition, multiplication and subtraction, *p* < 0.001, *p* < 0.001, *p* = 0.005, respectively. No other differences were found.

##### Accuracy Summary

Taken together, the changes in math fluency accuracy are the following: For addition, the main improvement occurs between the first and second grade, and then a plateau appears. For subtraction, the main improvement occurs between the first and second grade, and an additional improvement occurs between the second and fourth grade, and then a plateau appears. For multiplication, accuracy rates are high from the third grade on. For division, a significant change occurs between the third and fourth grade. However, for division, the accuracy rates in the plateau did not reach 90%, even in the sixth grade.

#### 3.4.3. RTs

##### General RTs

The analysis revealed a main effect of grade, *F* (5, 112) = 15.9, *p* < 0.001, *η_p_*^2^ = 0.42. Planned comparisons revealed that RTs in the first grade were lower and significantly different from those in all other grades, *p* < 0.001 for all comparisons. A trend analysis revealed a significant linear trend, *t* (112) = −8.0, *p* < 0.001; a quadratic trend, *t* (112) = 4.8, *p* < 0.001; and a cubic trend, *t* (112) = −2.8, *p* = 0.007.

##### Addition

The analysis revealed a main effect of grade, *F* (5, 110) = 15.3, *p* < 0.001, *η_p_*^2^ = 0.41. Planned comparisons revealed that RTs in the first grade were slower and significantly different from those in all other grades, *p* < 0.001 for all comparisons. A trend analysis revealed a significant linear trend, *t* (110) = −8.2, *p* < 0.001; a quadratic trend, *t* (110) = 4.9, *p* < 0.001; and a cubic trend, *t* (110) = −2.7, *p* = 0.008.

##### Subtraction

**Figure 9:**
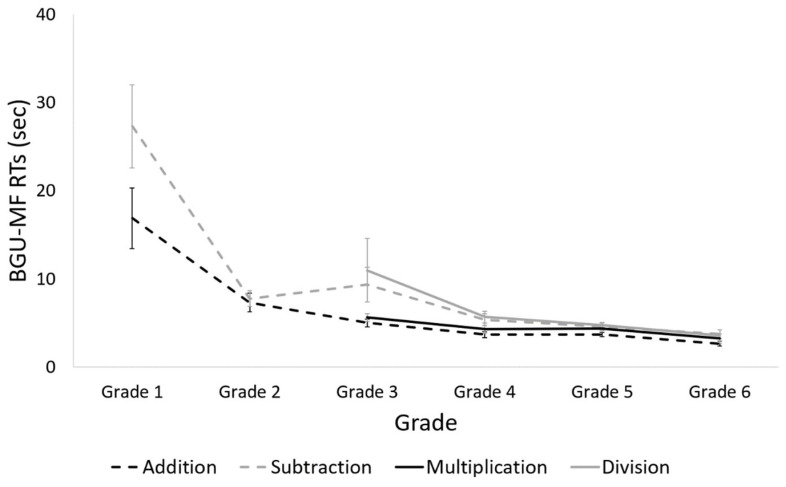
RTs in the BGU-MF, for every operation, in each grade.

The analysis revealed a main effect of grade, *F* (5, 108) = 15.0, *p* < 0.001, *η_p_*^2^ = 0.41. Planned comparisons revealed that RTs in the first grade were slower and significantly different from those in all other grades, *p* < 0.001 for all comparisons. RTs for the second grade were slower and significantly different from those in the fourth and sixth grade, *p* = 0.05. No other differences were found. A trend analysis revealed a significant linear trend, *t* (108) = −7.8, *p* < 0.001; a quadratic trend, *t* (108) = 4.8, *p* < 0.001; a cubic trend, *t* (108) = −3.3, *p* = 0.002; a quartic trend, *t* (108) = 2.6, *p* = 0.01; and a quintic trend, *t* (108) = −1.9, *p* = 0.06 (see Figure 9).

##### Multiplication

The analysis revealed a main effect of grade, *F* (3, 84) = 4.1, *p =* 0.009, *η_p_*^2^ = 0.13. Planned comparisons revealed that RTs in the third grade were slower and significantly different from those in the sixth grade, *p* = 0.004. No other differences were found. A trend analysis revealed a significant linear trend, *t* (84) = −3.3, *p* < 0.001.

##### Division

The analysis revealed a main effect of grade, *F* (3, 81) = 2.6, *p* = 0.05, *η_p_*^2^ = 0.09. RTs in the third grade were slower and significantly different from those in the sixth grade, *p* = 0.05. A trend analysis revealed a significant linear trend, *t* (81) = −2.6, *p* = 0.01.

##### Comparison between Operations

The analysis revealed a main effect of operation, *F* (3, 252) = 4.7, *p* = 0.003, *η_p_*^2^ = 0.05. Planned comparisons revealed no difference between RTs for addition and multiplication, and between subtraction and division. RTs for addition were faster and marginally significantly different than those for subtraction, *p* = 0.08, and were significantly different than those for division, *p* = 0.005. RTs for multiplication were faster and marginally significantly different than those for division, *p* = 0.07.

##### RT Summary

Taken together, the changes in math fluency accuracy are the following: For addition, the main reduction in RT occurs between the first and third grade, and then a plateau appears. For subtraction, a main reduction occurs between the first and second grade, and another reduction occurs between the second and fourth grade, and then a plateau appears. For multiplication and division, a slow change occurs from the third to sixth grade.

## 4. Discussion

The aim of the current study was to examine changes in mathematical fluency in grade one to grade six of primary school. To this end, we used the BGU-MF presented by Gliksman et al. [5]. In addition to math fluency performance in primary school, we examined the reliability and validity of the BGU-MF in children. Let us summarize our main results: (1) Students improve their math fluency during primary school in measures of the number of solved problems, accuracy rates, and RTs; (2) the changes that occur in math fluency differ by operation; (3) high correlations were found between first and second sessions of the BGU-MF, and between first and second halves of the test, in measures of the number of solved problems, accuracy rates, and RTs; (4) high correlations were found between manual (WJ) and computerized (BGU-MF) formats of math fluency, in measures of accuracy and the number of solved problems; (5) high correlations were found in math fluency as measured by the BGU-MF and as measured by arithmetic subtests of the WJ battery.

Regarding the changes that occur in math fluency across grade levels, we found that the number of solved problems increased and RTs decreased during all grades of primary school. Accuracy rates became high and stable for different operations at different stages: addition in the second grade, subtraction and multiplication in the fourth grade, and still not very high accuracy rates for division. Taken together, it seems that despite the fact that math facts are taught until the fourth grade, math fluency keeps improving even later. An educational implication of our result is that more practice is required to achieve proficiency in math facts after formal learning. Future studies should examine when, during adolescence in high school, performance becomes similar to that of adults.

Regarding addition and multiplication operations, our results are in line with the triple code model [22], as we found the performance of addition and multiplication math facts to be similar, and better in measures of accuracy rates and RTs, compared to subtraction and division math facts. The triple code model suggests that addition and multiplication math facts (but not subtraction or division) are related to the phonological code and that once proficiency in these operations is achieved, answers are retrieved from long-term memory and are not calculated. However, most studies related to the triple code model examined adults. Here we provide evidence that the use of the direct route of addition and multiplication math facts also occurs in children in primary school.

When, during primary school, does the change of strategy, from slower and serial strategies (i.e., counting), to faster, more efficient memory retrieval, occur? Previous studies reported that addition math facts are retrieved from memory starting in the third grade [12], and multiplication math facts are retrieved from memory starting in the fourth grade [23]. Note that we found similar results regarding accuracy rates and RTs for multiplication, but earlier (second grade) math facts retrieval from memory for addition. Future studies should examine when and if subtraction and division math facts are retrieved and not calculated. Using the BGU-MF might help explore this question, using the RT measure as a sign for the strategy of automatic retrieval.

Our results can contribute to the understating of the conceptual meaning of operations during development. Piaget [16] and later Siegler [15] suggested the principle of complementarity. According to this principle, a child does not fully understand the operation of addition or subtraction without understanding the relationship between them. That is, if 2 + 4 equals 6, then 6 − 2 must equal 4. The idea of complementary relations suggests that two complementary operations (e.g., addition and subtraction) should reach a plateau or automaticity at the same stage of development. In contrast, Baroody [17] argued that children do not necessarily relate to subtraction as the complementary operation of addition. He suggested that conceptual complementary relations may become automatic with practice. Our results support the view suggested by Baroody, as high accuracy rates for addition, probably indicating automaticity of this operation, were achieved before high accuracy rates for subtraction were achieved; and high accuracy rates for multiplication were achieved before high accuracy rates for division.

Our results of high correlations of performance between sessions suggest that the BGU-MF is a reliable tool to assess math fluency during primary school. Moreover, in general, no fatigue effects occurred during the test in any of the grades, but we did see improvement in the second half of the tests in measures of accuracy rates and RTs, specifically for first grade. These results expand previous reliability results of the BGU-MF in adults and support the suggestion that math fluency is a stable ability [5,6,21].

Our results of the high level of performance both in the manual and computerized formats of math fluency again strengthen the suggestion that math fluency is a stable ability, beyond format, and that the computerized BGU-MF examines math fluency validly. These results replicate and expand former results of adults who performed the computerized BGU-MF format and the manual WJ format [5]. Importantly, participants in all grades performed more problems in the manual format than in the computerized format. The gap between the formats derives from several reasons. First, the manual format of the WJ is more familiar to all students, and specifically, to children in the first grade. Second, only the BGU-MF includes the operation division, which has lower accuracy rates and longer RTs compared to other operations. Third, the order of the problems in the WJ format is that the first 60 problems are addition and subtraction problems, which are easier. Taking the difference with the familiarity of the formats into account, and the fact that first-grade children improve their performance in the second half of the BGU-MF test, we recommend that young students in primary school should perform the BGU-MF test twice. The first session should be considered as practice and should maybe be shorter than three minutes. The goal is familiarity with the computerized format procedure. The second session should be a full session (i.e., 3 min), whose results should be considered as the child’s proficiency score in math facts. Importantly, the computerized version has several advantages over the manual format: (1) Problems presented on the screen one by one allow attention to be focused on the specific problems; (2) the number of problems to be solved is not fixed in the computerized format but is only limited in time (3 min) in the BGU-MF. Thus, the computerized version may enable distinguishing children with high performance and overcome a ceiling effect; (3) the computerized tool is adaptive. Namely, the examiner may decide whether the test includes a specific operation or not, according to the curriculum, grade, and age of the student; (4) the computerized format provides RTs per problem and a summary per operation. Thus, a teacher or diagnostician may use the computerized tool to specifically examine the advantage or deficit in a specific problem or in one of the operations; (5) as mentioned, the BGU-MF includes all basic operations and thus is appropriate to test the proficiency of math fluency for students in all grades, as well as adults.

The performance in the BGU-MF test was correlated to performance in other WJ arithmetic tests. High accuracy in the WJ subtests was correlated with high accuracy in the BGU-MF and negatively correlated with RTs (see similar results [6]). Significant correlations were found with most subtests, but not with the quantitative concept’s subtest. The source of the correlation in performance might be that most subtests include simple calculations. For example, in order to complete a series of numbers, one should calculate the gap between them. In contrast, the quantitative concept requires symbol recognition, thus it differs in its demands from the other subtests. The correlation between WJ arithmetic tests and the BGU-MF test can also be explained by working memory resources. While calculating math facts, resources of working memory are occupied by the calculation process. When retrieval of math facts is achieved, working memory resources are free for other demands of the task, such as understanding the relevant problem in a text question [14]. From a wider perspective on mathematical cognition and its development, the current result supports the suggestion that proficiency with math facts is central to mathematical knowledge and achievements.

A limitation in our study should be considered. We examined participants only in their math fluency ability. Future studies should examine domain-general abilities, such as intelligence, reading abilities, and speed of processing, and explore whether they explain performance in the development of math fluency abilities.

To summarize, we present the BGU-MF computerized test to assess math fluency during primary school. The BGU-MF is a simple, adaptive, reliable, and valid tool. The BGU-MF shows the progress in acquiring mastery in mathematical facts during primary school years. It may be used to assess the proficiency of math facts, for diagnosis, or for training purposes. Importantly, about 10% of school children have math learning disabilities (MLD [24]), and deficits in the retrieval of math facts is one of the features of MLD [25,26]. Results of the BGU-MF might provide an early indication for MLD. Moreover, as each session in the BGU-MF test is different, it may be used for training as well. Finally, we recommend it for future research purposes.

## Figures and Tables

**Table 1:** Correlation coefficient (r) results between the BGU-MF test and the Woodcock–Johnson battery.

WJ Battery	BGU-MF		
	Numbers of Solved Problems	Accuracy Rates (%)	Average RT (s)
WJ math fluency (numbers of solved problems)	0.85 **	0.69 **	−0.59 **
WJ math fluency (accuracy rates)	0.52 **	0.76 **	−0.56 **
WJ applied problems (accuracy rates)	0.63 **	0.52 **	−0.29 *
WJ sequence completion (accuracy rates)	0.84 **	0.81 **	−0.60 **
WJ quantitative concept (accuracy rates)	0.26	0.11	−0.01
WJ calculation (accuracy rates)	0.56 **	0.57 **	−0.58 **

Note: BGU-MF—Ben-Gurion University Math Fluency test, WJ—Woodcock–Johnson, * *p* < 0.05; ** *p* < 0.01.

**Table 2:** Descriptive statistics for different grades and operations in the BGU-MF test.

	Grade 1	Grade 2	Grade 3	Grade 4	Grade 5	Grade 6
General						
Number of solved problems	3.8 (8.5)	15.6 (8.6)	22.2 (14.0)	31.2 (12.6)	31.6 (9.6)	41.1 (17.8)
Accuracy rates (%)	29.8 (31.1)	76.8 (23.3)	83.9 (15.5)	92.6 (6.4)	92.5 (7.4)	89.6 (13.4)
RTs (s)	14.1 (7.9)	8.1 (4.2)	6.9 (4.4)	4.5 (1.8)	4.3 (1.1)	3.3 (2.1)
Addition						
Accuracy rates (%)	31.5 (42.2)	82.8 (14.6)	88.5 (27.7)	95.0 (9.9)	95.6 (5.1)	92.0 (12.1)
RTs (s)	9.0 (3.8)	7.3 (4.3)	4.8 (1.9)	3.7 (1.6)	3.7 (1.2)	2.7 (1.4)
Subtraction						
Accuracy rates (%)	23.8 (31.6)	72.1 (33.8)	88.0 (16.4)	93.1 (8.1)	91.6 (11.8)	86.4 (23.7)
RTs (s)	22.6 (16.2)	8.0 (3.6)	7.2 (5.4)	5.4 (3.2)	4.6 (2.1)	3.2 (1.7)
Multiplication						
Accuracy rates (%)			85.4 (23.4)	94.7 (6.2)	95.4 (5.6)	90.2 (17.0)
RTs (s)			5.4 (2.2)	4.3 (2.2)	4.3 (1.8)	3.2 (2.5)
Division						
Accuracy rates (%)			68.2 (33.2)	87.5 (19.5)	87.2 (21.6)	88.4 (18.5)
RTs (s)			6.1 (2.3)	5.5 (3.3)	4.8 (1.4)	3.6 (3.1)

Note: Standard deviations in parentheses.

## Data Availability

Data will be available upon request from the first author. A link to the BGU-MF test appears in Section 2.2.1.

## Supplementary Materials

The following supporting information can be downloaded at: https://www.mdpi.com/article/10.3390/brainsci12030371/s1, Table S1: Addition and subtraction problems presented in the BGU-MF; Table S2: Multiplication and division problems presented in the BGU-MF; Figure S1: Number of solved problems in two halves of the BGU-MF test in each grade; Figure S2: Accuracy rates in two halves of the BGU-MF test in each grade; Figure S3: RTs in two halves of the BGU-MF test in each grade; Figure S4: Number of solved problems in the BGU-MF test and WJ Test in each grade; Figure S5: Accuracy rates in the BGU-MF test and the WJ test in each grade; Figure S6: Accuracy rates in the BGU-MF test and the WJ test by operation.
