# Supplementary material for: Math Fluency during Primary School
Source: Brain Sci. 2022 Mar 11;12(3):371. doi: 10.3390/brainsci12030371 (PMC8945962; doi:10.3390/brainsci12030371)
Supplement: Supplementary file 1 [file brainsci-12-00371-s001.zip › brainsci-1575646-supplementary.pdf]

Math Fluency During Primary School

Supplementary Materials

1. Pool of problems Presented in the BGU-MF

Addition and subtraction included problems with answer up to 20. For addition, each number in each row and column was presented as a first or second operator (e.g., 2 + 5 or 5 + 2), resulting 231 problems (see white problems in Table S1). For subtraction, the number presented in each row was presented as the first operator and the number presented in the column was presented and the second operator, resulting 231 problems (see gray problems in Table S1).

Table S1. Addition and subtraction problems presented in the BGU-MF.

Addition + Subtraction - 462 Problems.																					
	0	1	2	3	4	5	6	7	8	9	10	11	12	13	14	15	16	17	18	19	20
0	0	1	2	3	4	5	6	7	8	9	10	11	12	13	14	15	16	17	18	19	20
1	1	2	3	4	5	6	7	8	9	10	11	12	13	14	15	16	17	18	19	20	0
2	2	3	4	5	6	7	8	9	10	11	12	13	14	15	16	17	18	19	20	0	1
3	3	4	5	6	7	8	9	10	11	12	13	14	15	16	17	18	19	20	0	1	2
4	4	5	6	7	8	9	10	11	12	13	14	15	16	17	18	19	20	0	1	2	3
5	5	6	7	8	9	10	11	12	13	14	15	16	17	18	19	20	0	1	2	3	4
6	6	7	8	9	10	11	12	13	14	15	16	17	18	19	20	0	1	2	3	4	5
7	7	8	9	10	11	12	13	14	15	16	17	18	19	20	0	1	2	3	4	5	6
8	8	9	10	11	12	13	14	15	16	17	18	19	20	0	1	2	3	4	5	6	7
9	9	10	11	12	13	14	15	16	17	18	19	20	0	1	2	3	4	5	6	7	8
10	10	11	12	13	14	15	16	17	18	19	20	0	1	2	3	4	5	6	7	8	9
11	11	12	13	14	15	16	17	18	19	20	0	1	2	3	4	5	6	7	8	9	10
12	12	13	14	15	16	17	18	19	20	0	1	2	3	4	5	6	7	8	9	10	11
13	13	14	15	16	17	18	19	20	0	1	2	3	4	5	6	7	8	9	10	11	12
14	14	15	16	17	18	19	20	0	1	2	3	4	5	6	7	8	9	10	11	12	13
15	15	16	17	18	19	20	0	1	2	3	4	5	6	7	8	9	10	11	12	13	14
16	16	17	18	19	20	0	1	2	3	4	5	6	7	8	9	10	11	12	13	14	15
17	17	18	19	20	0	1	2	3	4	5	6	7	8	9	10	11	12	13	14	15	16
18	18	19	20	0	1	2	3	4	5	6	7	8	9	10	11	12	13	14	15	16	17
19	19	20	0	1	2	3	4	5	6	7	8	9	10	11	12	13	14	15	16	17	18
20	20	0	1	2	3	4	5	6	7	8	9	10	11	12	13	14	15	16	17	18	19
0	0	1	2	3	4	5	6	7	8	9	10	11	12	13	14	15	16	17	18	19	20

Multiplication and division included problems from the multiplication table. For multiplication, each number in the top row and left column was presented as a first or second operator (e.g., 2 x 5 or 5 x 2), including multiplication with the operator zero, resulting in 121 problems (see Table S2). For division, the number presented in the squares inside the table was the numerator, and the numbers presented in the top row or left column were the denominators (apart from zero as a denominator) (e.g., 20 : 4), resulting 110 problems (see Table S2).

Table S2. Multiplication and division problems presented in the BGU-MF.

Multiplication + Division - 231 Problems.											
	0	1	2	3	4	5	6	7	8	9	10
0	0	0	0	0	0	0	0	0	0	0	0
1	0	1	2	3	4	5	6	7	8	9	10
2	0	2	4	6	8	10	12	14	16	18	20
3	0	3	6	9	12	15	18	21	24	27	30
4	0	4	8	12	16	20	24	28	32	36	40
5	0	5	10	15	20	25	30	35	40	45	50
6	0	6	12	18	24	30	36	42	48	54	60
7	0	7	14	21	28	35	42	49	56	63	70
8	0	8	16	24	32	40	48	56	64	72	80
9	0	9	18	27	36	45	54	63	72	81	90
10	0	10	20	30	40	50	60	70	80	90	100

2. Split Test Reliability of BGU-MF

We compared performance between the first half of the BGU-MF test (0-90 seconds) and the second half (91-180 seconds). For each measure (i.e., number of correctly solved problems, accuracy rates and RT), a 2 (halves: first vs. second) × 6 (grades: first to sixth) ANOVA (analysis of variance) was carried out.

2.1. Number of Solved Problems

An ANOVA revealed no significant difference for halves, as the number of solved problems for the first and second halves were similar (12.7 for both halves), $F < 1$. A significant difference for grade was found, F (5, 237) = 34.3, $p < .001$, $\eta_p^2 = .4$. Planned

comparisons revealed that the number of solved problems for first grade was lower and significantly different from all other grades: p = .001 for second grade and p < .001 for third to sixth grade. The number of solved problems for second grade was lower and significantly different from fourth, fifth and sixth grade, p < .001 for all comparisons. The number of solved problems for third grade was lower and significantly different from fourth, fifth and sixth grade, p = .02, p = .02, and p < .001, respectively. In addition, for sixth grade, the number of solved problems was higher and significantly different from fourth, p = .02, and fifth grade, p = .02. An interaction between halves X grades was not significant, F (5, 237) = 1.5, p = .2, η_p^2 = .03 (see Figure S1).

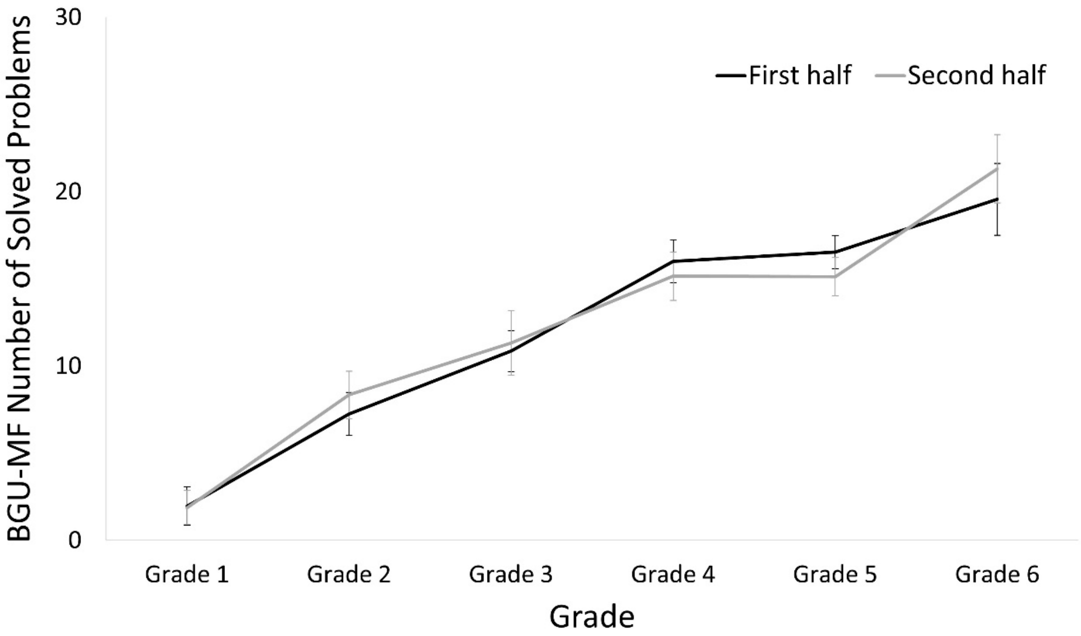

Figure S1. Number of solved problems in two halves of the BGU-MF test in each grade.

2.2. Accuracy Rates

The correlation between halves was significant, r = .64, p < .001. An ANOVA revealed a main effect of halves, as accuracy rates in the second half, 84.3% (SD = 24.5), were higher than in the first half, 79.0% (SD = 29.6), F (1, 222) = 6.1, p = .01, η_p^2 = .03. A main effect of

grade was found, F (5, 222) = 35.9, p < .001, η_p^2 = .45. Planned comparisons revealed that the accuracy rate for first grade was lower and significantly different from all other grades, p < .001 for all comparisons. Accuracy rate for second grade was lower and significantly different from fourth, fifth and sixth grades; p = .001, p = .001 *and* p = .05, respectively. No interaction between halves X grades was found, F (5, 222) = 1.6, p = .2, η_p^2 = .03 (see Figure S2).

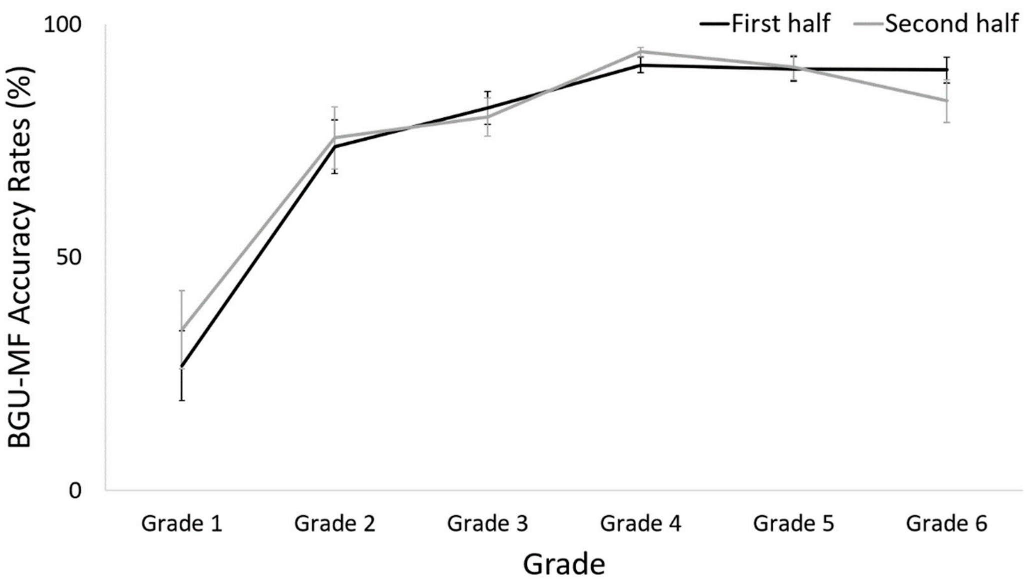

Figure S2. Accuracy rates in two halves of the BGU-MF test in each grade.

2.3. RTs

Analysis included only trials with a correct response. The correlation between halves was significant, r = .65, p < .001. An ANOVA revealed a main effect of halves, as RTs for the first half, 7.2 sec (SD = 10.2), were slower than for the second half, 5.4 sec (SD = 3.8), F (1, 207) = 41.1, p < .001, η_p^2 = .2. A main effect of grade was found, F (5, 207) = 27.6, p < .001, η_p^2 = .4. Planned comparisons revealed that RTs for first grade were slower and significantly different from all other grades, p < .001 for all comparisons. RTs for second grade were slower and significantly different from sixth grade, p = .02. An interaction between halves X

grades was found, *F* (5, 207) = 15.0, $p < .001$, $\eta_p^2 = .3$. Planned comparisons revealed that RTs differed between halves only in first grade, $p < .001$ (see Figure S3).

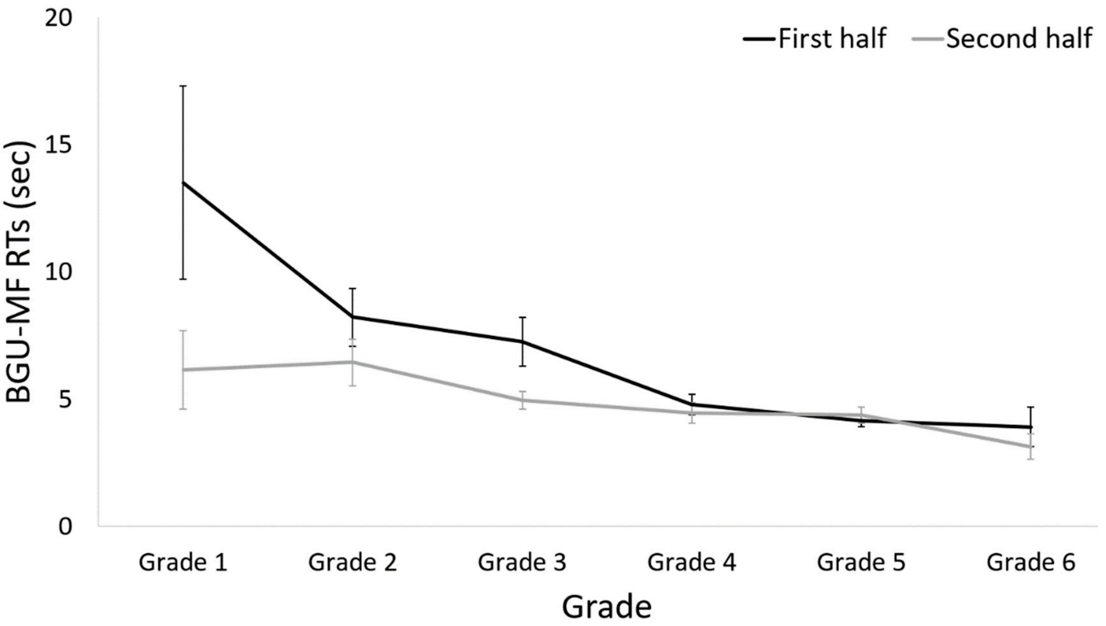

Figure S3. RTs in two halves of the BGU-MF test in each grade.

3. Validity Examination: Comparison between Math Fluency Formats

We compared the performance of math fluency using two formats—the BGU-MF computerized test and the WJ paper and pencil math fluency test. We performed a 2 (formats: BGU-MF vs. WJ) × 6 (grades: first to sixth) ANOVA, with the number of problems and accuracy rates as dependent variables.

3.1. Number of Solved Problems

Analysis revealed a main effect of format, as the number of solved problems was higher in the WJ format than in the BGU-MF format, *F* (1, 116) = 529.9, $p < .001$, $\eta_p^2 = .82$. A main effect of grade was found, *F* (5, 116) = 31.6, $p < .001$, $\eta_p^2 = .6$. Planned comparison revealed that the number of solved problems for first grade was lower and significantly different from all other grades; $p = .005$ for second grade, $p < .001$ for third to sixth grade. The number of solved problems for second grade was lower and significantly different from fourth, fifth and sixth grade, $p < .001$ for all comparisons. In addition, the number of solved

problems for sixth grade was higher and significantly different from third to fifth grade; *p* < .001 for third grade, *p* = .003 for fourth grade, and *p* = .01 for fifth grade). A significant Interaction between format x grade was found, *F* (5, 116) = 10.9, *p* < .001, η_p^2 = .31. Planned comparisons revealed a significant difference between formats for second to sixth grades, *p* < .001 for all comparisons. A marginally significant difference was found in first grade, *p* = .06.

Figure S4. Number of solved problems in the BGU-MF test and WJ test in each grade.

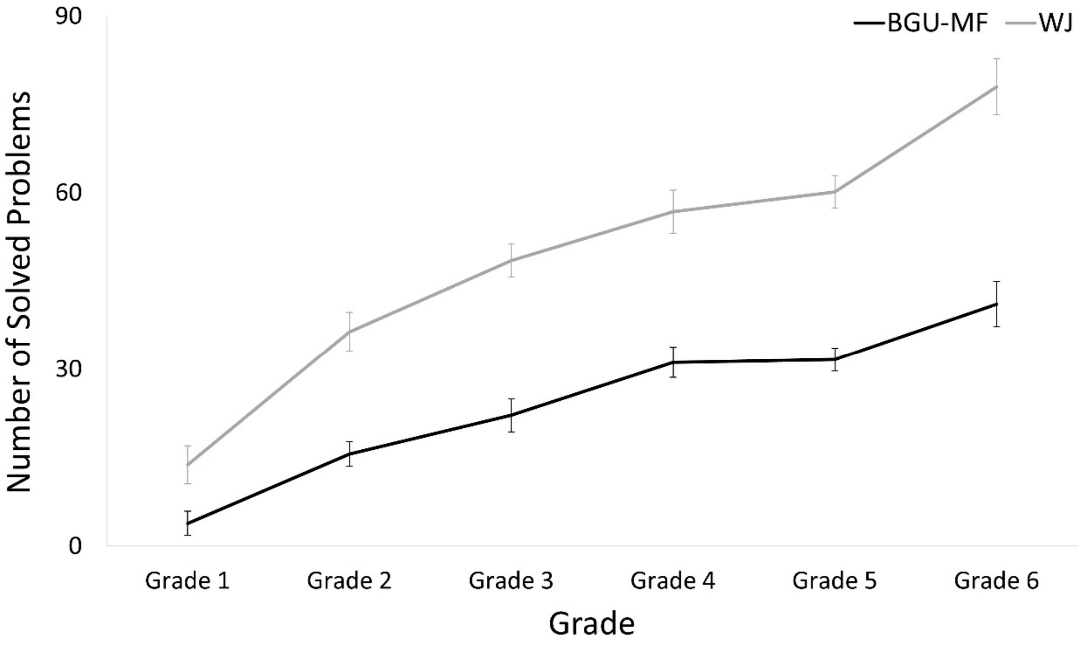

3.2. Accuracy Rates

Analysis revealed a main effect of format, as accuracy rates for the WJ were higher than for the BGU-MF, *F* (1, 116) = 101.7, *p* < .001, η_p^2 = .47. A main effect of grade was found, *F* (5, 116) = 48.6, *p* < .001, η_p^2 = .68. Planned comparisons revealed that accuracy rates for first grade were lower and significantly different from all other grades, *p* < .001. A significant Interaction between format x grade was found, *F* (5, 116) = 10.9, *p* < .001, η_p^2 =

.32. Planned comparisons revealed that the difference between formats was significant in first grade, $p < .001$, second grade, $p = .001$, and third grade, $p = .002$ (see Figure S5).

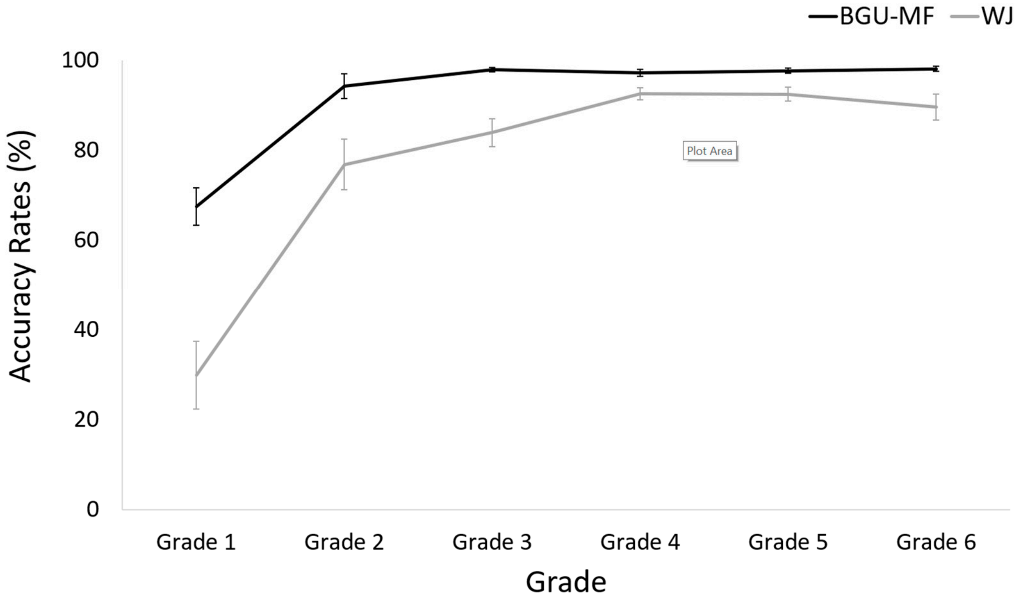

Figure S5. Accuracy rates in the BGU-MF test and the WJ test in each grade.

4. Format and Operation

We compared the performance of math fluency using the two formats with the different operations. A 2 (formats: BGU-MF vs. WJ) × 3 (operations: addition, subtraction, and multiplication) ANOVA was carried out, with accuracy rates as the dependent variable. The analysis revealed a main effect of format, F (1, 88) = 1,242.8, $p < .001$, $\eta_p^2 = .93$, and a main effect of operation, F (2, 176) = 318.3, $p < .001$, $\eta_p^2 = .78$. Planned comparisons revealed the accuracy rates differed for all operations, $p < .001$ for all comparisons. A significant interaction between format and operation was found, F (2, 176) = 289.9, $p < .001$, $\eta_p^2 = .77$. Planned comparisons revealed a difference between formats for all the operations, $p < .001$ for all comparisons (see Figure S6).

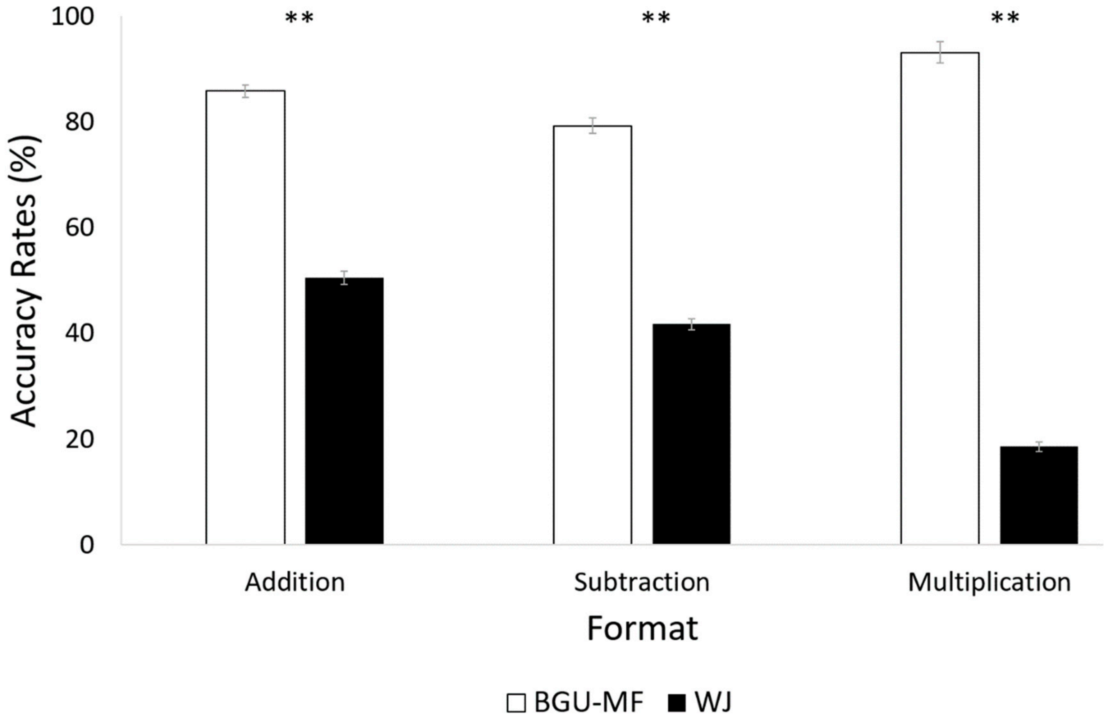

Figure S6. Accuracy rates in the BGU-MF test and the WJ test by operation. $^{**}p < .01$.
